# Assessment of Antioxidant Activity of Spray Dried Extracts of *Psidium guajava* Leaves by DPPH and Chemiluminescence Inhibition in Human Neutrophils

**DOI:** 10.1155/2014/382891

**Published:** 2014-04-13

**Authors:** M. R. V. Fernandes, A. E. C. S. Azzolini, M. L. L. Martinez, C. R. F. Souza, Y. M. Lucisano-Valim, W. P. Oliveira

**Affiliations:** Faculty of Pharmaceutical Sciences of Ribeirão Preto, Laboratory of R&D on Pharmaceutical Processes-LAPROFAR, University of São Paulo, Avenue do Café S/N, BL. Q, 14040-903 Ribeirão Preto, SP, Brazil

## Abstract

This work evaluated the physicochemical properties and antioxidant activity of spray dried extracts (SDE) from *Psidium guajava* L. leaves. Different drying carriers, namely, maltodextrin, colloidal silicon dioxide, Arabic gum, and **β**-cyclodextrin at concentrations of 40 and 80% relative to solids content, were added to drying composition. SDE were characterized through determination of the total phenolic, tannins, and flavonoid content. Antioxidant potential of the SDE was assessed by two assays: cellular test that measures the luminol-enhanced chemiluminescence (LumCL) produced by neutrophils stimulated with phorbol myristate acetate (PMA) and the DPPH radical scavenging (DPPH∗ method). In both assays the antioxidant activity of the SDE occurred in a concentration-dependent manner and showed no toxicity to the cells. Using the CL_lum_ method, the IC_50_ ranged from 5.42 to 6.50 µg/mL. The IC_50_ of the SDE ranged from 7.96 to 8.11 µg/mL using the DPPH^•^ method. *Psidium guajava* SDE presented significant antioxidant activity; thus they show high potential as an active phytopharmaceutical ingredient. Our findings in human neutrophils are pharmacologically relevant since they indicate that *P. guajava* SDE is a potential antioxidant and anti-inflammatory agent in human cells.

## 1. Introduction

Currently, there is a growing interest in herbal products with antioxidant properties, which have the potential to protect the body against free radical damage and degenerative diseases. Several studies have been conducted to find new sources of compounds capable to inactivate free radicals generated by metabolic pathways in human tissues and cells, produced mainly by human neutrophils [1]. These cells are the most abundant circulating leukocytes and are the first line of defense against microorganisms, cells infected by viruses, and tumor cells [2].

Standardized plant preparations are generally administered in the form of liquid extracts, viscous products, and powders resulting from the drying and comminuting of plant material or by drying an extract [3]. Among these options, the tendency in the pharmaceutical industry is to use dry extracts. Products in solid form show significant advantages over conventional liquid forms, including chemical, physical-chemical, and microbiological stability; ease of standardization; higher concentration of active compounds; ease of transportation; less space for product storage; less risk of microbial contamination; and the capacity to transform powders into different solid pharmaceutical forms. Transforming a plant extract into dry extract is also widely used in developing herbal medicinal products [4].

Drying processes generally used in the preparation of dry extracts spray drying, spouted bed drying, and freeze-drying include. Due to the complex composition of herbal extracts, the drying method must be selected based on its physical and chemical properties, thermal stability, and the intended use of same [5].

Several authors have used the spray drying technique for standardization of active phytopharmaceutical ingredients and thereby guaranteeing the efficacy, quality, and safety of the product [6]. In general, the powders obtained by this drying method have good reconstitution characteristics and low water activity and are suitable for transport and storage [7]; therefore they are frequently used as intermediate and final products, particularly in solid dosage forms. Various drying carriers are used for drying, including maltodextrin, modified starch, cyclodextrins, Arabic gum, microcrystalline cellulose, and colloidal silicon dioxide, in order to improve process performance and product quality. Drying carriers can be used individually or as blends and the proportions established are specific to each case considering both physical and chemical properties of extract to be dried and the drying technology to be used [5].

The aim of this work was to produce and characterize spray dried extracts from* Psidium guajava* leaves using different drying carriers and evaluate their antioxidant potential; through their inhibitory effect on the generation of reactive oxygen species (ROS) by stimulated neutrophils and by ROS scavenging properties in a cell-free system using the DPPH radical methodology.

*Psidium guajava* L. (Myrtaceae), popularly known as guava, is originally from Central and South America and is cultivated in all tropical and subtropical countries [8]. Extracts and metabolites of this plant, particularly from the leaves and fruits, possess useful pharmacological activities, such as antispasmodic and antimicrobial properties, and have been used in the treatment of diarrhea. Other reported uses include its function as a hypoglycemic, antitussive, anti-inflammatory, and antioxidant agent, thereby reinforcing its traditional use [9]. This plant has been used in traditional and folk medicine in many parts of the world. In Brazil, it is inserted in the list of plant species selected for study by the Research Program on Medicinal Plants (*Programa de Pesquisas de Plantas Medicinais*, PPPM) and included in the National Report of Medicinal Plants of Interest to the Public Health System [10].

## 2. Material and Methods

### 2.1. Chemicals

The chemicals used in this study are as follows: methanol (A1085.01.BJ), aluminum chloride (C1036.01.AG), ethanol (A1084.01.BJ), and hydrochloric acid (A1028.01.BJ; Labsynth, Diadema, SP, Brazil); sodium tungstate-2-hydrate (230), triton X-100, and phosphomolybdic acid (443; Vetec Química Fina Ltda, Duque de Caxias, RJ, Brazil); gallic acid monohydrate (27645-250G-R), 1,1-diphenyl-2-picrylhydrazyl (DPPH^•^; D9132), luminol (5-amino-2,3-dihydro-1,4-phthalazinedione; A8511-5G), phorbol-12-myristate-13-acetate (PMA; P8139), and Trypan blue (T6146-25G; Sigma-Aldrich, St. Louis, MO, USA); gelatin (microbiological grade; 214320, Difco, Laboratories, Detroit, MN, USA); dimethyl sulfoxide (DMSO; 3317275) (Merck-Schuchardt, Hohenbrunn, DE); “Kit” to determine lactate dehydrogenase enzyme (LDH Liquiform; 86-2/30—Labtest Diagnostica, Lagoa Santa, MG, Brazil). Drying carriers used are colloidal silicon dioxide (Aerosil 200, Evonik Degussa, Hanau, Germany), maltodextrin (MOR-REX 1910, Corn Products do Brasil), **β**-cyclodextrin (Kleptose; Roquette, Lestrem, FR), and gum Arabic (Encapsia; NEXIRA do Brasil, São Paulo, SP, Brazil).

### 2.2. Plant Material

*Psidium guajava *leaves (Pedro Sato cultivar) were collected in February 2011 at the farm and industry* Casa da Goiaba*, in Lavras, MG (21°13′32.9′′ S, 44°59′06.05′′ W and altitude of 883 m). The plant material was identified by Professor Dr. Marcelo Polo and the exsiccate was deposited in the Herbarium of the Federal University of Alfenas (UALF-01505). The fresh leaves were dried in an oven with circulating air at a temperature of 45°C until a constant weight was achieved and ground in a Marconi knife mill (MA mod. 680), using 20 mesh (833 *μ*m).

### 2.3. Extraction of Bioactive Compounds

The dried and ground leaves were extracted by dynamic maceration under constant stirring for 60 min, using ethanol 70% (v/v) at 50°C. The plant/solvent ratio was set at 1 : 10 (w/v). The extraction solution obtained was filtered and concentrated in a rotary evaporator (Fisatom, mod. 802) at 50°C under vacuum of 650 mmHg, until solid contents reached 10.7%. The extractive solution and concentrated extract were analyzed for total phenolics, total tannins, and total flavonoid contents.

### 2.4. Spray Drying

Drying carriers were added to the concentrated extract (8%, wet base) before spray drying. The drying carriers used and the codes of the dried extracts (DE) obtained are presented in Table 1.

A benchtop spray dryer (model SD 05, Lab-Plant, UK) with a drying chamber, measuring 215 mm in diameter and 500 mm in height, with a concurrent flow regime was used in the drying runs. The equipment has as peripherals a suspension supply system consisting of a peristaltic pump, a dual fluid atomizer (1 mm diameter inlet orifice), and an air compressor; a supply system for the drying gas, consisting of a compressor and an air filter; a temperature control system for the drying gas; and a powder product collection system (cyclone).

The operating parameters were set according to previous studies carried out by our research group [11–13]: inlet air drying temperature, *T*_gi_ = 150°C; drying air flow, *W*_*g*_ = 60 m^3^/h; extract feed flow rate, *W*_susp_ = 4 g/min; atomizing air pressure, *P*_atm_ = 1.5 bar; and atomizing air flow rate, *W*_atm_ = 15 lpm. During the drying experiments, the room temperature and relative humidity (%) were monitored by using a digital hygrometer Minipa MTH, 1361.

Dryer performance was evaluated by determining the product recovery (*R*) and thermal efficiency of the system (*η*).  Table 2 presents the equations used in these determinations [3].

### 2.5. Characterization of the Spray Dried Product

The spray dried product was characterized by quantification of the product moisture content, water activity, particle size, solubility in water, total polyphenols, tannins and flavonoid contents, and antioxidant activity. The methods used in these characterizations are presented in the following.

#### 2.5.1. Product Moisture Content and Water Activity

Product moisture content was determined by Karl Fischer's volumetric method (Karl Fischer-model 870 KF Titrino plus, Metrohm, Switzerland).

The water activity of the dried extract was determined using the equipment Aqua Lab 4TEV (Decagon Devices, USA), using the dew-point sensor at 25°C immediately after the drying process. The results are expressed as the average of three determinations.

#### 2.5.2. Solubility in Water

The water solubility of the dried products was determined according to Cano-Chauca et al. [14], with some modifications. Samples of 100 mg of powder were exactly weighted and added to 10 mL of distilled water, being vigorously stirred for 10 min in a magnetic stirrer. The samples were centrifuged for 5 min at 3000 rpm in a laboratory centrifuge (Fanem, Model 206, São Paulo, Brazil). An aliquot of approximately 2 mL of the supernatant was transferred to a previously weighted Petri dish. The solid concentration was determined by the oven drying method previously described. Results were expressed as grams of soluble powder per 100 g of water (*n* = 3).

#### 2.5.3. Particle Size and Distribution

Particle size distribution was determined using the image analysis by optical microscopy. Samples of the dried extracts were dispersed on the surface of microscopy laminas and images of the powders were obtained at 50x magnification using an Olympus microscope (model BX60MIV) coupled with a digital camera (model 3.2.0, SPOT Insight, Diagnostic Instruments) and analyzed using Image-Pro Plus 7.0 software. The measurements obtained were used to determine the mean particle diameter and the cumulative frequency distribution [15]. The process was repeated using at least three laminas and a minimum of 1000 particles were measured to ensure the reliability and repeatability of the method [16].

#### 2.5.4. Determination of Total Polyphenols, Tannins, and Flavonoids Contents

Total polyphenols (TP) and tannins (TT) contents were quantified using the Folin-Denis method, which involves the reduction of phosphomolybdic-phosphotungstic acid by phenolics in alkaline medium resulting in an intense blue color, being the absorvance measured by UV-vis spectrophotometry at a wavelength of 750 nm after a reaction time of 2 min [3, 17]. The results are expressed as gallic acid equivalents per gram of extract or gram of plant material using an analytical curve. The samples were analyzed in triplicate.

The total flavonoids content (TF) was quantified using a spectrophotometric method based on the displacement of the wavelength at 425 nm following the addition of 0.5% AlCl_3_ (w/v) with a reaction time of 30 min. Absorbance was measured at 425 nm using a UV/VIS HP 8453 spectrophotometer (Agilent Technologies, Waldbronn, Germany). Total flavonoids content was expressed in milligrams of quercetin per gram of dried product, as determined by an analytical curve. The samples were analyzed in triplicate.

#### 2.5.5. Evaluation of Antioxidant Activity

Evaluation of the antioxidant activity (*A*_*A*_) of original extractive solution, concentrated extract, and the spray dried products was determined by two distinct* in vitro* assays that quantified free radical scavenging activity using the 1,1-diphenyl-2-picrylhydrazyl radical [18] and the chemiluminescence amplified by luminol (CL_lum_) in a cell medium [19].

*(1) Antioxidant Activity Using the DPPH* ^•^ *Method*. DPPH radical scavenging activity was measured using the Blois method [20], described by Georgetti et al. [18]. Ten *μ*L of solutions of the concentrated extract and of the spray dried products were assayed at different concentrations (10, 20, 40, 50, 60, and 80 *μ*g/mL). These were added to a reaction mixture containing 1.0 mL of 0.1 M acetate buffer (pH 5.5), 1.0 mL ethanol, and 0.5 mL of DPPH^•^ 0.250 mM ethanolic solution. The change in absorbance was measured after 20 min at 517 nm at 25°C. The results were expressed as IC_50_ (*μ*g/mL), which reflects the substrate concentration required to produce a 50% reduction in DPPH^•^, and are presented as the mean and standard deviation obtained in triplicate measurements.

*(2) Assays in Cell Models*

*Isolation of Human Neutrophils*. Individuals healthy volunteers (*n* = 10) of both sexes, aged between 18 and 40 years old, who were not using medications, were recruited in accordance with the protocol approved by the Ethics Committee on Human Research (CEP) of the Faculty of Pharmaceutical Sciences of Ribeirao Preto (FCFRP) of the University of São Paulo (CEP/FCFRP number 108/2011). The blood was collected from volunteers by venipuncture using Alséver solution, pH 6.1 (v/v), as an anticoagulant. Neutrophils were isolated according to the Henson method [21], modified by Lucisano and Mantovani [22]. Cell viability was determined by the Trypan blue exclusion assay, which yielded cell preparations with viability greater than 87%.

*(3) Evaluation of ROS Production by Chemiluminescence*. Antioxidant activity in the cellular system evaluated by chemiluminescence (CL) was performed according to Lucisano-Valim et al. [19]. CL measurements were performed in a final volume of 0.5 mL. The chemiluminescent probe, luminol (0.28 mM), and 5 *μ*L of different concentrations of the extracts evaluated or 20% DMSO (control) was added to 250 *μ*L of neutrophil solution (1 × 10^6^ cells/mL) and the mixture was incubated for 3 min at 37°C. The cells were then stimulated with 50 *μ*L of 10^−7^ M phorbol-12-myristate-13-acetate (PMA). The chemiluminescence produced by the cells was monitored for 20 min in a luminometer (Auto Lumat LB 953 EG & G Berthold, Bad Wildbad, Baden-Württemberg, Germany), in which the light output was recorded as cpm (counted photons per min). The integrated area values (area under the curve) were calculated from curves of CL versus time. The percentage inhibition of CL promoted by each sample was calculated using the following formula: 100 − [(AS/AC) × 100], where AS is the integrated area of each sample evaluated at each concentration, and AC is the integrated area of the control sample. IC_50_ values (substrate concentration that inhibits 50% of CL) were calculated by nonlinear regression and used to compare the inhibitory effects. Three independent experiments were performed in duplicate.

*(4) Cytotoxicity Study*. Evaluation of the toxicity of the concentrate extract and dried products was performed to check whether the modulatory activities obtained on neutrophils were due to the toxic effects on the cells. Analyses were performed as described by Kabeya et al. [23]. Aliquots of the neutrophil suspension (1 × 10^6^ cells/mL) were incubated for 20 min at 37°C in Hanks solution (negative control), 0.2% Triton X-100 (positive control), dimethylsulfoxide (DMSO) (control solvent, 0.25 or 1%), or the samples under evaluation, at concentrations of 5 and 50 *μ*g/mL dissolved in DMSO; the final reaction volume was 1 mL. Following centrifugation (755 ×g, 10 min, 4°C), the cell sediments were resuspended in Hanks solution containing 0.1% gelatin and cell viability was assessed by the Trypan blue exclusion assay, based on counts of 200 cells.

The activity of lactate dehydrogenase (LDH) released in the supernatant was calculated after measuring the decrease in absorbance at 340 nm for 3 min at 37°C using a Liquiform LDH kit. Total lysis of the cells (positive control) was achieved with 0.2% (v/v) Triton X-100. Toxicity was evaluated in three independent experiments measured in duplicate.

### 2.6. Statistical Analysis

The statistical analysis of the experimental data was carried out with the aid of SAS software, version 9.0. The Kruskal-Wallis test was also proposed, followed by Dunn's posttest to reveal statistical differences between groups [24, 25], using a significance level of 5%. The results were obtained with the aid of the software R 2.14.0, using the PGIRMESS library of functions [26].

Analysis of variance (ANOVA) followed by the Tukey test were carried out for the experimental data of the assays involving cells, with the aid of the GraphPad Prism software (version 3.0 for Windows, GraphPad Software Inc., San Diego, CA, USA).

## 3. Results and Discussion

### 3.1. Characterization of the Extraction Solution and Concentrated Extract

The extractive solution and concentrated extracts were analyzed for solids (*C*_*s*_), density (*ρ*), pH, alcohol content, total flavonoids (TF), total polyphenols (TP) and total tannins (TT) contents, and antioxidant activity (*A*_*A*_) using the DPPH^•^ method (Table 3).

Concentration of the extractive solution was performed to increase the concentration of solids to approximately 10.7%, more suitable for drying [16]. Table 3 indicates that no significant loss of active substances or antioxidant activity occurred during the concentration step. It can be seen that both extractive solution and concentrated extract show remarkable free scavenging potential, presenting an IC_50_ lower than 4.0 *μ*g/mL.

### 3.2. Spray Drying

The properties of the feed composition were modified by the addition of different drying carriers, and their effects on drying performance, physical and chemical product properties, and antioxidant activity are presented in the following.

### 3.3. Drying Performance

Table 4 shows the experimental results obtained for spray drying performance, in terms of outlet drying gas temperature (*T*_go_), product recovery (*R*), and process thermal efficiency (*η*). The experimental results of product recovery are consistent with the ones reported by Tonon et al. [27], which shows *R* between 34 and 55%, when maltodextrin was used as drying carrier. These values were influenced by changes in the process operating conditions, such as inlet gas temperature (138–202°C), maltodextrin concentration (10–30%), and feed flow rate of feed composition (5–25 g/min). According to Goula and Adamopoulos [28], one source of material loss occurs in the cyclone. Since the cyclone used had a cut diameter near 3.9 *μ*m, lower particles would be carried out with the exhaust gas. Oliveira et al. [16] evaluated the powder losses in both spray and spouted bed drying systems, presenting results almost similar to the ones obtained in this work.

Thermal efficiency is a parameter commonly used to optimize the drying process since it is linked with the energy consumption. In this study, this parameter was estimated by the energy balance of the drying gas at the inlet and outlet of the dryer. It can be seen in the data presented in Table 4 that the drying carriers used did not present significant effect on spray drying thermal efficiency. Souza et al. [3] showed that the thermal efficiency tended to be increased conversely with the flow rate of feed composition, with the other variables maintained constant.

### 3.4. Physical Properties of **P. guajava** Spray Dried Extracts

Table 5 presents the physical properties of the spray dried powders of* Psidium guajava* engineered with different drying carriers.

Product moisture content (*X*_KF_) from 3.93 to 5.82 was obtained, which can be considered as adequate assuming the maximum moisture content recommended by the U.S. Pharmacopoeia [29] for dry extracts of medicinal plants (≤5%). The values obtained in this work are in agreement with those reported by Gallo et al. [30], which ranged from 2.41 (±0.08) to 4.72 (±0.28)%.

The water activity (*a*_*w*_) of dried product is an important parameter, which is linked with microbiological and chemical product stability. Since it is a determining factor for values of *a*_*w*_ lower than 0.5 and does not permit the growth of microorganisms [30, 31]. As can be seen in Table 5, in this work, the spray dried powders showed *a*_*w*_ values lower than 0.5, which favors the physicochemical stability of the products.

Regarding the water solubility, the spray dried products containing maltodextrin and aerosil (MA 80), maltodextrin, aerosil and gum Arabic (MDEA 80), and *β*-cyclodextrin (*β*-CD) were quite soluble and did not show statistically significant differences (*P* > 0.05) between them. These results are in agreement with the ones reported by Cano-Chauca et al. [14], who used maltodextrin as a drying carrier for mango juice and obtained solubility values greater than 90%. This trend was also observed for spray dried preparations of* Bidens pilosa* [12]. Analysis of the results revealed that the most water soluble dried products were obtained from compositions containing *β*-cyclodextrin and the maltodextrin-aerosil mixture as drying carriers.

It can be seen in Table 5 that the spray dried powders showed mean particle ranging from 12.1 to 15.8 *μ*m, therefore within the range expected for spray dried powders, from 5 to 150 *μ*m [32]. The results did not evidence significant effects of the feed composition on the mean particle size. Similar results were reported by Souza et al. [3], during spray drying of* Rosmarinus officinalis* in presence of colloidal silicon dioxide and maltodextrin, using the same setup and similar drying conditions, which obtained powder particles with mean diameters ranging from 9 to 13 *μ*m. In general, the product diameter is affected by several processing and feed composition factors, such as drying operating conditions, type and concentration of drying carriers, and dryer configuration, including the powder collecting system.

#### 3.4.1. Total Polyphenols, Tannins, and Flavonoids Contents

**Figure 1:**
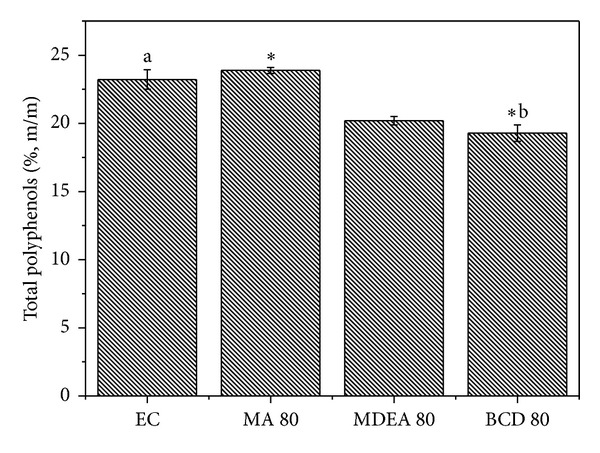
Comparisons between the total polyphenols from the concentrated extract and spray dried extracts using the Kruskal-Wallis test (*P* < 0.05). ^∗ab^Statistical difference determined by Dunn's posttest.

**Figure 2:**
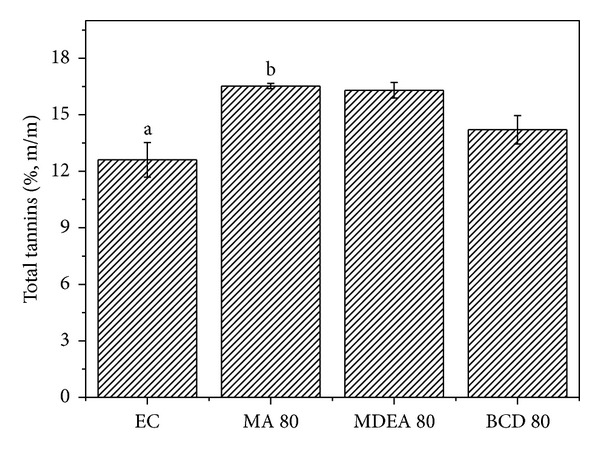
Comparisons between the total tannins from the concentrated extract and spray dried extracts using the Kruskal-Wallis test (*P* < 0.05). ^ab^Statistical difference determined by Dunn's posttest.

**Figure 3:**
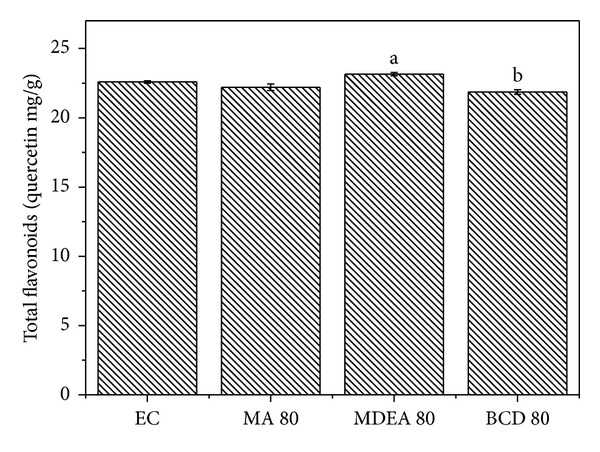
Comparisons between the total flavonoids from the concentrated extract and spray dried extracts using the Kruskal-Wallis test (*P* < 0.05). ^ab^Statistical difference determined by Dunn's posttest.

One way to standardize herbal products involves controlling the concentration of market compounds that may or may not be related to their biological activity. The compounds selected as markers in this study have been reported as responsible for the antioxidant, antimicrobial, and antidiarrheal activities of the plant* Psidium guajava* [9]. Comparisons between the concentrations of chemical markers (total flavonoids, total polyphenols, and total tannins) of the concentrated extract with the spray dried ones yielded similar values, while revealing some statistical difference between the groups (Figures 1, 2, and 3).

Among the drying compositions, the maltodextrin and aerosil (MA 80) mixture and the formulation prepared only with *β*-cyclodextrin (**β**CD 80) were the ones with the lowest reduction of the contents of chemical markers. These results are consistent with the recent work of Cortés-Rojas and Oliveira [12], who compared several drying carriers and observed that the drying composition based on *β*-cyclodextrin showed the highest flavonoid concentration. In the pharmaceutical field, cyclodextrins are useful due to their interaction with drug molecules to form inclusion complexes. During the formation of a complex, the physicochemical and biological properties of the pharmaceutical can be altered, including improving its solubility and its physical and chemical stability [33]. Maltodextrin is often used in the spray drying process because of its low cost, high water solubility and low viscosity in solution [34]. Tonon et al. [27] studied the effect of maltodextrin DE10 and reported that this drying carrier retained around 80% of the anthocyanidins present in açai juice, following spray drying, which is in agreement with results observed in this work, in which the maltodextrin and aerosil formulation (MA 80) retained most of the marker compounds.

#### 3.4.2. Antioxidant Activity of* P. guajava *Extract and Spray Dried Products

The antioxidant activity of extractive solution, concentrated extract, and spray dried powders was determined by two different methodologies: DPPH^•^ method and evaluation of ROS production in a cell medium measured by chemiluminescence assay.

*(1) Antioxidant Activity by DPPH* ^•^ *Method*.  Table 6 shows the experimental results of antioxidant activity measured by the DPPH^•^ method of the spray dried powders and of concentrated extract (control). The quercetin is a natural antioxidant flavonoid used as a reference standard. IC_50_ values (concentration required to inhibit 50% of the oxidative reaction) were used to present the experimental data.

The percentage of maximum inhibition of the samples evaluated showed similar values, ranging from 85.9 to 88.5%. A slight decrease in antioxidant activity occurred, characterized by higher IC_50_ values for the spray dried product compared with the concentrated extract. Statistically significant differences (*P* ≤ 0.05) were only observed for the samples of concentrated extract and MDEA 80. Georgetti et al. [13] reported maximum inhibition for DPPH^•^ of 52.8% (±0.97%) for spray dried soybean extracts with maltodextrin as drying carrier, while the concentrated extract showed approximately 75% of maximum DPPH^•^ inhibition. The authors also used colloidal silicon dioxide and starch as spray drying carriers and verified a correlation between antioxidant activity and the total polyphenols content of the extract. The results suggest that chemical composition and antioxidant activity are affected by the drying temperature. Thus, compounds capable of providing a synergistic effect for antioxidant activity can be damaged or eliminated by the drying process, contributing to a reduction in the antioxidant effectiveness of the dried product [35].

**Figure 4:**
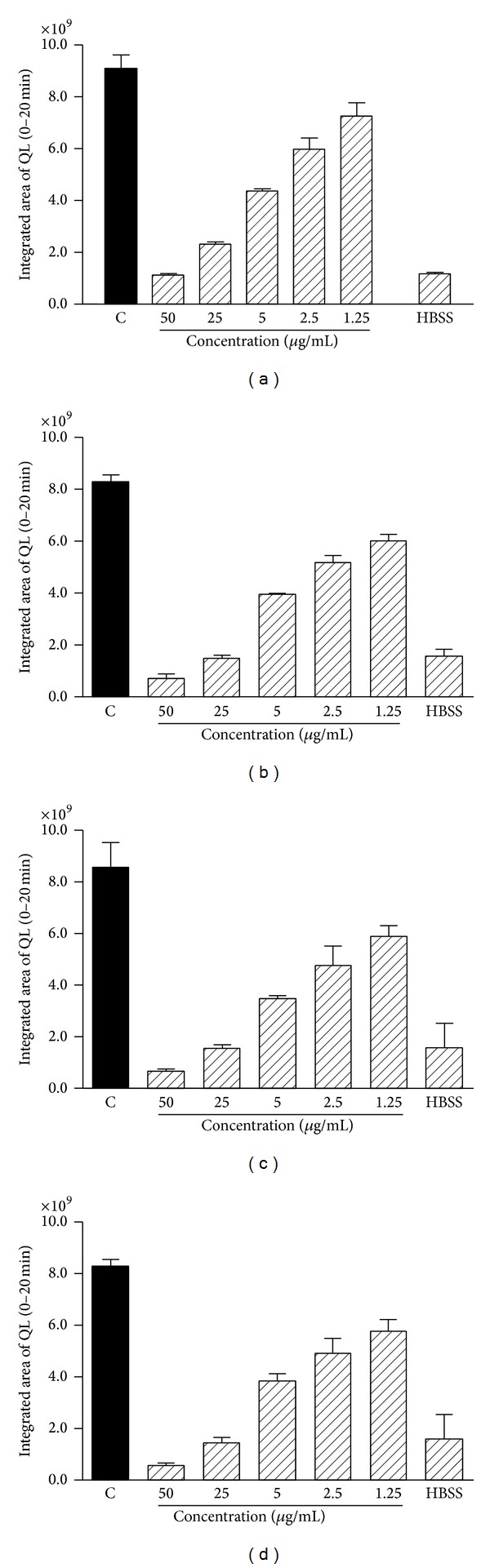
Modulatory effect of different concentrations of the extracts studied on ROS production in human neutrophils stimulated with PMA, determined by luminol-dependent chemiluminescence assay. (a) Conc. Ext. (concentrated extract) (b) MA 80, (c) MDEA 80, and (d) *β*CD 80. Data expressed as the integrated area of CL 0–20 min. The figure is representative of three independent experiments with similar profiles performed in duplicate.

*(2) Evaluation of ROS Production by Chemiluminescence*. Assays involving CL have been widely used for monitoring ROS generated by enzymes, cells, and tissues due to its sensitivity and reproducibility [36]. In this study, total ROS production by neutrophils stimulated with phorbol-12-myristate-13-acetate (PMA) was measured by chemiluminescence assay with a luminol probe (CL_lum_). PMA is a soluble stimulus synthetic and known for its tumor promoting action. Neutrophils activated by this stimulus produce luminol-dependent chemiluminescence profiles immediately following the addition of the stimulus that persistent for over 20 min [36], as confirmed in this study. In all the assays, the concentrated extract and spray dried products were solubilized in 25% DMSO, obtaining a final solvent concentration of 0.25%. Laboratory studies have shown this DMSO concentration does not interfere with CL_lum_ production (data not shown). The concentrated extract and spray dried product showed a concentration-dependent inhibitory effect of CL_lum_ on PMA stimulated neutrophils (Figure 4).

A comparative study of the inhibitory effect of CL on the concentrated extract and spray dried products was conducted based on the calculation of the IC_50_ values (Table 7). The results obtained showed that the concentrated extract and spray dried product evaluated were able to modulate ROS production by neutrophils activated with PMA in a concentration-dependent manner.

Several studies using different plant extracts have reported a modulatory effect on stimulated neutrophil samples. In the work of Paula et al. [36] a modulatory effect of an extract of the fruit pulp of* Tamarindus indica* was observed on neutrophils stimulated with PMA and this effect was also concentration dependent. The authors reported that the extract was more effective as an inhibitor of neutrophil oxidative metabolism induced by PMA compared with other stimuli. Studying the antioxidant activity of an extract of* Calendula officinalis*, Braga et al. [1] also evaluated the inhibitory effects in human neutrophils CL_lum_ activated by PMA. They verified that the extract exerted its anti-ROS activity in a concentration-dependent manner and that it presented a significant effect even at low concentrations (1.60 *μ*g/mL).

The results obtained here showed that both the concentrated extract and the spray dried products promoted an inhibitory effect on luminol-dependent chemiluminescence. This observed effect suggests a ROS scavenger effect, which was confirmed by* in vitro* evaluation of free radical scavenging activity (DPPH^•^). Since this study was conducted using crude extract, with no investigation of isolated compounds, it was not possible to elucidate which of the metabolites present in the extract are responsible for the effects observed. Despite this limitation, studies with crude extracts are important because the biological activities of a natural product are not usually due to a single compound, but to the synergism between various substances [37]. No statistically significant differences were verified between the concentrated extract and spray dried products, which indicates that neither the drying process nor the drying carrier significantly interfered with the inhibitory effect presented by the samples studied.

*(3) Evaluation of the Cytotoxicity of Psidium guajava*. The cytotoxicity of the concentrated extract and the spray dried products was verified by two assays: the Trypan blue exclusion assay and determination of lactate dehydrogenase (LDH) enzyme activity. The aim was to evaluate whether the modulator effect presented by the samples on the neutrophils was associated with diminished neutrophil function, determined by decreasing CL_lum_, or whether it was related to a toxic effect on the cells of these samples. Initially, the concentrated extract was assessed for toxicity against neutrophils at concentrations of 5 *μ*g/mL (data not shown) and 50 *μ*g/mL. Later, the same test was performed with the spray dried product and concentrated extract at a concentration of 50 *μ*g/mL. In the Trypan blue exclusion assay, in the presence of the extracts, the cells obtained viabilities greater than 93%, and using the kit to determine LDH enzyme activity, the percentage release of the enzyme relative to the positive control (Triton X-100) was low compared with the negative control values. These results suggest that all the extracts evaluated showed no cytotoxic activity on neutrophils using these assays (Table 8).

## 4. Conclusion

Standardized dried extracts from* P. guajava *leaves containing various drying carriers were successfully engineered by spray drying. Product recoveries of around 57% were verified during the drying runs; however, the operating parameters were fixed in order to permit quantifiable comparisons between the different feed compositions. No significant changes in the concentrations of marker compounds (total polyphenols, tannins, and flavonoids) were observed.

Product properties in terms of moisture content and water activity were inside the recommended ranges, in order to minimize product spoilage due to chemical degrading reactions or microbial growth. The product also presented high water solubility (from 83 to 94%), and an increase in solubility was observed when maltodextrin was used as drying carrier. The concentrated extract and the spray dried extracts presented pronounced free radical scavenging activity in both the DPPH method and in assay involving PMA-stimulated human neutrophils. These findings provide information concerning the potential* in vivo* modulatory effect of the product on the effect or functions of human neutrophils.

In conclusion, analysis of the results obtained confirmed the effective antioxidant activity of spray dried* Psidium guajava* extracts indicating his potential to be used as an active phytopharmaceutical ingredient for food and pharmaceutical uses.

## Figures and Tables

**Table 1:** Drying carriers added to feed compositions.

Samples code	Drying Aids	Ratio (%)
MA 80	Maltodextrin DE10 : aerosil	7 : 1
MDEA 80	Maltodextrin DE10 : gum Arabic : aerosil	5 : 2 : 1
*β*CD 80	*β*-Cyclodextrin	8

**Table 2:** Equations used to determine the *spray dryer* performance.

Parameter	Equation	Unit	Equation
Product recovery (*R*)	$R=\frac{\text{Mc}\cdot (1-X_{p})}{W_{s}\cdot C_{s}\cdot \theta }\times 100$	(%) g/g	(1)

Thermal efficiency (*η*)	$\eta =\frac{\left(T_{\text{gi}}-T_{\text{go}}\right)}{\left(T_{\text{gi}}-T_{\text{amb}}\right)}\times 100$	(—)	(2)

Mc: mass collected (g); *X*_*p*_: product moisture content (g/g); *W*_*s*_: flow rate of feed composition (g/min); *C*_*s*_: concentration of total solids (g/g), *θ*: processing time (min); *T*_gi_: inlet gas temperature (°C); *T*_go_: outlet gas temperature (°C); *T*_amb_: environment temperature (°C).

**Table 3:** Physicochemical characterization of the extractive solution (ES) and of concentrated extract (CE) of *P. guajava*.

Samples	*C*_*s*_ (%)	*ρ* (g/cm^3^)	pH (—)	Alcoholic grade (°GL)	TF (mg/m db)	TP (% m/m db)	TT (% m/m db)	DPPH^•^^1^ (µg/mL)
SE	3.27 (0.03)	0.89 (0.00)	5.94 (0.02)	70	23.55 (0.01)	26.29 (0.03)	11.31 (0.02)	3.94 (0.10)
CE	10.7 (0.12)	0.93 (0.00)	4.90 (0.07)	0	22.52 (0.01)	23.17 (0.07)	12.61 (0.10)	3.34 (0.11)

*C*_*s*_: concentration of solids; *ρ*: density; TF: total flavonoids; TP: total polyphenols; TT: total tannins; db: dry base; ^*1*^antioxidant activity by the DPPH^•^ method, expressed as IC_50_. Values are presented as the mean (standard deviation).

**Table 4:** Spray dryer performance.

Samples	*T*_go_ (°C)	*T*_amb_ (°C)	RH (%)	*R* (%)	*η* (%)
MA 80	109.0	28.9	42.1	51.38	33.9
MDEA 80	100.0	21.2	46.6	59.63	38.8
**β**CD 80	102.0	20.2	58.1	61.48	37.0

*T*_go_: outlet gas temperature; *T*_rm_: room temperature; RH: relative humidity; *R*: product recovery; *η*: thermal efficiency.

**Table 5:** Physical characterization of *Psidium guajava* spray dried extracts.

Samples	*X*_*p*_ (%)	*a*_*w*_ (—)	Water solub. (%)	*d*_p_* (µm)
MA 80	4.34 (0.18)	0.16 (0.03)	94.4 (1.9)	13.3 (12.0)
MDEA 80	5.82 (0.36)	0.19 (0.03)	87.7 (5.7)	12.1 (12.7)
**β**CD 80	3.93 (0.05)	0.18 (0.03)	83.4 (1.6)	15.8 (16.4)

*X*_*p*_: product moisture (Karl-Fischer titration); *a*_*w*_: water activity *d_*p*_: mean diameter of the particles (deviation of particle size distribution, not the mean particle size). Values are presented as the mean (standard deviation).

**Table 6:** Antioxidant activity of the dry extracts of *P. guajava *using the DPPH^•^ method.

Samples	IC_50_ (µg/mL)	% Inhibition
MA 80	8.11 (0.05)	88.5 (0.5)
MDEA 80	9.76 (0.21)*	85.9 (0.9)
CD 80	7.96 (0.18)	86.1 (0.8)
Conc. Ext.	3.34 (0.11)*	87.3 (0.6)
Quercetin	0.96 (0.01)	86.2 (0.9)

*P* < 0.05. *Statistical difference determined by Dunn's posttest.

Values are presented as the mean (standard deviation).

**Table 7:** Inhibitory effect of the concentrated extract and spray dried extracts of *Psidium guajava *on CL_lum_ produced by neutrophils stimulated with PMA.

Conc. (µg/mL)^a^	Percentage inhibition (%)^b^			
	Conc. Ext.	MA 80	MDEA 80	**β**CD 80
1.25	22.40 (10.77)	26.46 (1.56)	26.36 (4.73)	27.98 (4.92)
2.5	35.23 (6.69)	32.0 (6.79)	37.54 (7.89)	34.6 (8.60)
5.0	48.25 (5.22)	51.52 (8.68)	50.72 (9.63)	47.62 (9.11)
25.0	74.37 (4.02)	77.4 (8.55)	77.27 (8.40)	77.29 (10.0)
50.0	86.39 (3.02)	87.56 (5.99)	88.76 (5.56)	88.6 (7.95)

**I** **C**_50_	6.03 (1.77)	5.54 (2.24)	5.42 (2.85)	6.50 (3.60)
**n**	3	3	3	3

**Quercetin** **I** **C**_50_	1.67 (1.57)			

^a^Final concentration of the concentrated extract and spray dried product evaluated; ^b^percentage inhibition of CL_lum_ produced by neutrophils stimulated with PMA.

Values are presented as the mean (standard deviation).

**I** **C**_50_: sample concentration that promotes 50% inhibition of chemiluminescence.

**n**: number of independent experiments performed in duplicate.

IC_50_: *P* = 0.08 (no statistically significant difference was determined by Dunn's posttest).

**Table 8:** Analysis of the cytotoxicity of concentrated extract and spray dried extracts of *Psidium guajava* on stimulated neutrophils.

Samples	Viable cells (%)^c^ Trypan blue	LDH^d^ activity (U/mL)	Liberated LDH (% positive control)^e^
Positive control^a^	—	0.1516 (0.00)	100
Negative control^b^	96.5 (0.71)	0.0126 (0.01)	8.40 (5.85)
DMSO control (0.2%)	97.0 (0.71)	0.0166 (0.002)	11.10 (1.55)
Conc. Ext.	94.00 (2.83)	0.0137 (0.02)	7.58 (8.76)
**MA 80**	94.2 (3.18)	0.0158 (0.01)	10.53 (3.68)
**MDEA 80**	96.7 (1.06)	0.0153 (0.001)	10.20 (0.47)
**β** **CD 80**	93.2 (1.06)	0.0166 (0.00)	11.07 (0.19)

Values are presented as the mean (standard deviation). ^a^1 × 10^6^ neutrophils/mL lysed with 0.2% Triton X-100; ^b^1 × 10^6^ neutrophils/mL incubated with Hanks; DMSO: solvent control; ^c^% of viable cells, determined by Trypan blue exclusion assay, based on counts of 200 cells; ^d^LDH enzyme activity, evaluated by the Liquiform (Labtest Diagnóstica) LDH kit; ^e^percentage of LDH liberated in the supernatant compared with neutrophils completely lysed with Triton X-100.

## References

[1] Braga PC, Dal Sasso M, Culici M (2009). Antioxidant activity of *Calendula officinalis* extract: inhibitory effects on chemiluminescence of human neutrophil bursts and electron paramagnetic resonance spectroscopy. *Pharmacology*.

[2] Selvatici R, Falzarano S, Mollica A, Spisani S (2006). Signal transduction pathways triggered by selective formylpeptide analogues in human neutrophils. *European Journal of Pharmacology*.

[3] Souza CRF, Schiavetto IA, Thomazini FCF, Oliveira WP (2008). Processing of *Rosmarinus officinalis* Linne extract on spray and spouted bed dryers. *Brazilian Journal of Chemical Engineering*.

[4] Oliveira WP, Souza CRF, Kurozawa LE, Park KJ, Woo MW, Mujundar AS, Daud WRW (2010). Spray drying of food and herbal products. *Spray Drying Technology*.

[5] Oliveira WP, Freitas LAP, Freire JT, Freire JT, Silveira AM, Ferreira MC (2012). Drying of pharmaceutical products. *Transport Phenomena in Particulate Systems*.

[6] Bott RF, Labuza TP, Oliveira WP (2010). Stability testing of spray- and spouted bed-dried extracts of *Passiflora alata*. *Drying Technology*.

[7] Kha TC, Nguyen MH, Roach PD (2010). Effects of spray drying conditions on the physicochemical and antioxidant properties of the Gac (*Momordica cochinchinensis*) fruit aril powder. *Journal of Food Engineering*.

[8] Alves PM, Leite PHAS, Pereira JV (2006). Antifungal activity of the extract of *Psidium guajava* Linn. (“goiabeira”) upon leavens of *Candida* of the oral cavity: an *in vitro* evaluation. *Brazilian Journal of Pharmacognosy*.

[9] Gutiérrez RMP, Mitchell S, Solis RV (2008). *Psidium guajava* a review of its traditional uses, phytochemistry and pharmacology. *Journal of Ethnopharmacology*.

[10] BRASIL Relação Nacional de Plantas Medicinais de Interesse ao SUS, (RENISUS). http://portal.saude.gov.br/portal/arquivos/pdf/RENISUS.pdf.

[11] Souza CRF, Oliveira WP (2006). Powder properties and system behavior during spray drying of *Bauhinia forficata* link extract. *Drying Technology*.

[12] Cortés-Rojas DF, Oliveira WP (2012). Physicochemical properties of phytopharmaceutical preparations as affected by drying methods and carriers. *Drying Technology*.

[13] Georgetti SR, Casagrande R, Souza CRF, Oliveira WP, Fonseca MJV (2008). Spray drying of the soybean extract: effects on chemical properties and antioxidant activity. *LWT—Food Science and Technology*.

[14] Cano-Chauca M, Stringheta PC, Ramos AM, Cal-Vidal J (2005). Effect of the carriers on the microstructure of mango powder obtained by spray drying and its functional characterization. *Innovative Food Science and Emerging Technologies*.

[15] Souza CRF, Oliveira WP (2005). Spouted bed drying of *Bauhinia forficata* link extract: the effects of feed atomizer position and operating conditions on equipment performance and product properties. *Brazilian Journal of Chemical Engineering*.

[16] Oliveira WP, Bott RF, Souza CRF (2006). Manufacture of standardized dried extracts from medicinal Brazilian plants. *Drying Technology*.

[17] Folin O, Denis W (1912). On phosphotungstic-phosphomolybdic compounds as color regents. *Journal of Biological Chemistry*.

[18] Georgetti SR, Casagrande R, Moura-de-Carvalho Vicentini FT, Verri WA, Fonseca MJV (2006). Evaluation of the antioxidant activity of soybean extract by different in vitro methods and investigation of this activity after its incorporation in topical formulations. *European Journal of Pharmaceutics and Biopharmaceutics*.

[19] Lucisano-Valim YM, Kabeya LM, Kanashiro A (2002). A simple method to study the activity of natural compounds on the chemiluminescence of neutrophils upon stimulation by immune complexes. *Journal of Pharmacological and Toxicological Methods*.

[20] Blois MS (1958). Antioxidant determinations by the use of a stable free radical. *Nature*.

[21] Henson PM (1971). The immunologic release of constituents from neutrophil leukocytes. I. The role of antibody and complement on nonphagocytosable surfaces or phagocytosable particles. *Journal of Immunology*.

[22] Lucisano YM, Mantovani B (1984). Lysosomal enzyme release from polymorphonuclear leukocytes induced by immune complexes of IgM and of IgG. *Journal of Immunology*.

[23] Kabeya LM, da Silva CHTP, Kanashiro A (2008). Inhibition of immune complex-mediated neutrophil oxidative metabolism: a pharmacophore model for 3-phenylcoumarin derivatives using GRIND-based 3D-QSAR and 2D-QSAR procedures. *European Journal of Medicinal Chemistry*.

[24] Pagano M, Gauvreau K (2004). *Princípios de Bioestatística*.

[25] Hollander M, Wolfe DA (1999). *Nonparametrics Statistics Methods*.

[26] R Development Core Team (2011). *R Foundation For Statistical Computing*.

[27] Tonon RV, Brabet C, Hubinger MD (2008). Influence of process conditions on the physicochemical properties of açai (*Euterpe oleraceae* Mart.) powder produced by spray drying. *Journal of Food Engineering*.

[28] Goula AM, Adamopoulos KG (2004). Spray drying of tomato pulp: effect of feed concentration. *Drying Technology*.

[29] USP XXX (2007). *United States Pharmacopeia*.

[30] Gallo L, Llabot JM, Allemandi D, Bucalá V, Piña J (2011). Influence of spray-drying operating conditions on *Rhamnus purshiana* (Cáscara sagrada) extract powder physical properties. *Powder Technology*.

[31] Araújo RR, Teixeira CCC, Freitas LAP (2010). The preparation of ternary solid dispersions of an herbal drug via spray drying of liquid feed. *Drying Technology*.

[32] Favaro-Trindade CS, Pinho SC, Rocha GA (2008). Review: microencapsulation of food ingredients. *Brazilian Journal of Food Technology*.

[33] Stella VJ, He Q (2008). Cyclodextrins. *Toxicologic Pathology*.

[34] Turchiuli C, Fuchs M, Bohin M (2005). Oil encapsulation by spray drying and fluidised bed agglomeration. *Innovative Food Science and Emerging Technologies*.

[35] Cousins M, Adelberg J, Chen F, Rieck J (2007). Antioxidant capacity of fresh and dried rhizomes from four clones of turmeric (*Curcuma longa* L.) grown *in vitro*. *Industrial Crops and Products*.

[36] Paula FS, Kabeya LM, Kanashiro A (2009). Modulation of human neutrophil oxidative metabolism and degranulation by extract of *Tamarindus indica* L. fruit pulp. *Food and Chemical Toxicology*.

[37] Verpoorte R, Choi YH, Kim HK (2005). Ethnopharmacology and systems biology: a perfect holistic match. *Journal of Ethnopharmacology*.

